# Nose-to-Brain Delivery of Antioxidants as a Potential Tool for the Therapy of Neurological Diseases

**DOI:** 10.3390/pharmaceutics12121246

**Published:** 2020-12-21

**Authors:** Maria Cristina Bonferoni, Giovanna Rassu, Elisabetta Gavini, Milena Sorrenti, Laura Catenacci, Paolo Giunchedi

**Affiliations:** 1 Department of Drug Sciences, University of Pavia, 27100 Pavia, Italy; cbonferoni@unipv.it (M.C.B.); milena.sorrenti@unipv.it (M.S.); laura.catenacci@unipv.it (L.C.); 2 Department of Chemistry and Pharmacy, University of Sassari, 07100 Sassari, Italy; grassu@uniss.it (G.R.); eligav@uniss.it (E.G.)

**Keywords:** antioxidants, neurological diseases, nose-to-brain, nanomedicine, brain targeting

## Abstract

Oxidative stress has a key role in the pathogenesis of neurodegenerative disorders such as Alzheimer’s, Parkinson’s, and Huntington’s diseases and can be an important cause of the damages in cerebral ischemia. Oxidative stress arises from high levels of reactive oxygen species (ROS). Consequently, on this rational base, antioxidants (many of natural origin) are proposed as potential drugs to prevent ROS noxious actions because they can protect the target tissues from the oxidative stress. However, the potential of antioxidants is limited, owing to the presence of the blood–brain barrier (BBB), which is difficult to cross with a consequent low bioavailability of the drug into the brain after systemic (intravenous, intraperitoneal, oral) administrations. One strategy to improve the delivery of antioxidants to the brain involves the use of the so-called nose-to-brain route, with the administration of the antioxidant in specific nasal formulations and its passage to the central nervous system (CNS) mainly through the olfactory nerve way. In the current literature, many examples show encouraging results in studies carried out in cell cultures and in animal models about the potential neuroprotective effects of antioxidants when administered through the nose. This review concerns the nose-to-brain route for the brain targeting of antioxidants as a potential tool for the therapy of neurological diseases.

## 1. Introduction

Oxidative stress is due to the lack of equilibrium between the biochemical processes of production of the reactive oxygen species (ROS) and the cellular antioxidant cascade [1,2]: the formation of ROS that exceeds the natural antioxidant barrier determines an imbalance of the pro-/antioxidant events [2,3].

Oxidative stress has a key role in the pathogenesis of neurodegenerative diseases such as Alzheimer’s, Parkinson’s, Huntington’s diseases, and amyotrophic lateral sclerosis, [4,5]. In humans, the central nervous system (CNS) is particularly sensitive to oxidative damage owing to its high oxygen consumption (about 20% of the total body basal oxygen consumption) [6]. It has been reported that samples from Parkinson’s disease brains have shown reduced levels of redox enzymes, catalase and superoxide dismutase [7]. Furthermore, ROS lead to misfolding and the aggregation of proteins, and to DNA/RNA oxidation [8], and it is significant that all the neurodegenerative diseases have a similarity in the aggregation of specific misfolded proteins in the CNS [9].

It is also known that oxidative stress can play an important role in transient cerebral ischemia, which is characterized by the interruption of blood flow followed by reperfusion: during this second phase, oxygen acts as substate of a series of enzymatic oxidation reactions resulting in an increase of oxidant species [10]. Therefore, the pharmacologic modification of oxidative damage has been proposed as a potential strategy for stroke therapy [11].

All these considerations strongly encourage the use of antioxidants as a possible instrument to prevent ROS action on the neurological targets: several preclinical studies reported that antioxidants diminish the oxidative stress and improve cognitive impairments in the case of Alzheimer’s disease [2]. On the other hand, several drugs used in the therapy of Alzheimer’s disease have some antioxidant properties that probably contribute to their efficacy [2].

However, despite the rational basis of this approach, there are discordant reports in the literature about the real efficacy of these molecules for the therapy of neurological diseases, and nowadays, the clinical efficacy of antioxidants is still limited and has given disappointing outcomes [8,12]. Although different antioxidants (many of natural origin) have been investigated as potential drugs, none of them has shown to be efficacious in a large-scale controlled study [13].

Among the reasons of this unclear situation, a relevant role can be attributed to the problem of the difficult crossing of the blood–brain barrier (BBB) that determines a low and irregular bioavailability of the drug into the brain after systemic administration. The BBB is characterized by selective permeability due to its complex cellular system and to the astrocytes [14]. This barrier is formed by microvascular endothelial cells able to selectively control the entry of endogenous/exogenous substances into the brain [14].

Therefore, a big challenge for the therapy of neurological diseases is to reach the efficient delivery of drugs to the brain.

**Figure 1:**
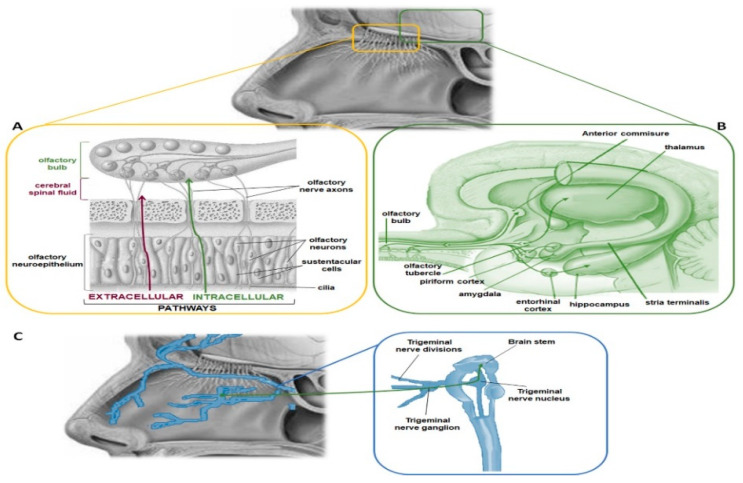
Nose-to-brain pathways. (**A**) The transport of molecules from the nose to the brain occurs via intracellular pathway through the olfactory nerves and via extracellular pathway through the paracellular space between olfactory neurons and supporting cells of the olfactory neuroepithelium. If the molecules diffuse through the paracellular space, they reach the extracellular fluid of the nasal lamina propria that is continuous with the cerebrospinal fluid of the subarachnoid space. (**B**) Following internalization by the olfactory neurons, the molecules are directly transported to the central nervous system (CNS) and, in particular, in the piriform cortex, the amygdala and olfactory tubercle. (**C**) The intracellular mechanism involves also the trigeminal nerves, which originates in the pons of the brainstem and, thus, can transport the internalised drug in this CNS area.

In the early 1990s, the possibility of overcoming the BBB was discovered by means of the non-invasive intranasal route [15]. The intranasal route involves two pathways (Figure 1): one intracellular and the other extracellular [16]. The intracellular pathway begins in the olfactory region with the endocytosis process carried out by the olfactory cells, which is followed by the axonal transport to the synapses of the olfactory bulb, where the drug is exocytosed [16]. This trans-synaptic process is repeated by the olfactory neurons; thus, the drug is distributed to other regions of the brain. In the extracellular pathway, the drug is transported directly into the cerebral spinal fluid by passing through the paracellular spaces across the nasal epithelium and through the perineural space to the subarachnoid space of the brain [16]. The olfactory region is in the upper part of the nasal cavity, it has a surface area of about 2.5–10 cm^2^ in humans [17], and it is directly connected to the brain via the olfactory nerves [18]. Therefore, the nasal administration allows direct pathways to the brain, by which the drugs can penetrate in the different regions of the brain [16,18]. Furthermore, the mechanisms of drug transport depend on many other factors such as drug properties, characteristics of the delivery system, and physiological conditions [18]. Anyway, the mechanisms of transport of the drug from the nasal cavity to the brain are still under study.

Additionally, the nose-to-brain route allows the administration of reduced drug doses, while obtaining a therapeutic response equivalent to, if not better than, the oral drug route, mainly because both the drug enzymatic degradation in the gastrointestinal tract and the hepatic first-pass effect are avoided. For this reason, the nasal route can be used to administer drugs with problems of oral bioavailability. Furthermore, it is a non-invasive and safe route for many patients [14].

This approach created new therapeutic opportunities to treat neurodegenerative disorders including examples of intranasal drug delivery in humans [19]. These studies can be considered the rational base for the development of nasal formulations for brain targeting.

For these reasons, innovative formulations are studied to deliver the drugs using the nose-to-brain route: the carrier systems include specific drug delivery systems such as, for instance, polymeric nanoparticles (for example made by biodegradable polymers) [18,20,21], solid lipid nanoparticles (SLNs) [22], and nanoemulsions [23,24]. The present review concerns the use of the nose-to-brain route for the administration of antioxidants as a potential tool for the therapy of neurological diseases.

## 2. Antioxidants Used as Potential Tool for the Therapy of Neurological Diseases

The greatest number of papers found in this overview concerned two antioxidants of natural origin, curcumin (Table 1) and resveratrol (Table 2). However, other molecules with antioxidant activity have also been investigated (Table 3). All the studies involved animal models and/or cell cultures; no clinical studies have been developed yet.

### 2.1. Curcumin

**Figure 2:**
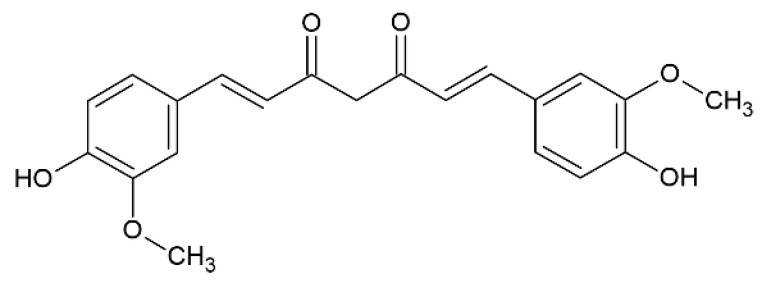
Curcumin.

Curcumin (Figure 2) is a potent antioxidant polyphenol obtained from rhizomes of turmeric (*Curcuma longa* L.), which is a curry spice commonly used in India, exhibiting remarkable antioxidant and anti-inflammatory properties that can be beneficial in the treatment of oxidative stress [48].

It is claimed that curcumin inhibits free radicals and reduces lipid peroxidation [48]. It is also reported that curcumin determines the inhibition of cyclooxygenase, phospholipase, and of the chemotaxis of monocytes that arrest neuronal inflammation [48]. Curcumin has also a strong action of chelator of iron and copper, which are redox-active metal ions that are involved in the inflammation process and in the progression of Alzheimer’s disease [48]. Furthermore, curcumin inhibits the process of plaque aggregations of amyloid β(Aβ) and destabilizes the Aβ fragments [49,50]. Ganguli et al. showed that US population has 4.4 times more prevalence of Alzheimer’s disease in the age of 70–79 years with respect to Indian population and correlates this to the use of curry spice in India [51].

Despite these biological properties, the therapeutic use of curcumin is limited owing to its short half-life, low water solubility, and consequent poor oral bioavailability [52].

Desai et al. prepared cocrystal micelles containing curcumin, designed for intranasal delivery, as a possible treatment of Alzheimer’s disease [25]. Pharmaceutical cocrystals are single-phase crystalline structures constituted by a drug and a coformer in a specific stoichiometric ratio. The pharmacological activity of the drug in the cocrystals is maintained and, at the same time, owing to the conformer, a modification/modulation of its physical properties occurs. In particular, by using a hydrophilic conformer, cocrystals can improve the water solubility of poorly soluble drugs. Desai developed cocrystals containing curcumin and made with “smart” hydrophilic functional coformers with additional antioxidant activity. This class of “antioxidant-hydrophilic” coformers were used with a dual goal: the enhancement of the aqueous solubility curcumin and at the same time the improvement of the antioxidant activity. Hildebrand solubility parameter (δ) [53] was employed as a screening parameter for the choice of the possible materials to be used as coformers. “δ” is an indicator of solvency behavior and the materials with similar “δ” result in solvation and miscibility. The tested materials were citric acid, ascorbic acid, malic acid, fumaric acid, sorbic acid, succinic acid, tartaric acid, quercetin, and a so-called “coformer A”. Succinic acid and “coformer A” were chosen as the most suitable on the basis of the “δ” value. The curcumin cocrystals were prepared using a ball mill method, and the process was optimized and then validated by a design of experiment approach, using a 3^2^ factorial design. Curcumin cocrystal micellar formulations were developed with a process of preparation of the micelles that involved the use of surfactants such as Tween 80. The final formulations had the aspect of a clear, homogenous light-yellow liquid that was then packed in a nasal spray. The cocrystal micelles showed a globule size of about 29 nm. In vitro drug release tests were carried out using a dialysis bag method in 100 mL of phosphate buffer (pH 6). Curcumin cocrystals showed more than 3-fold enhancement solubility than plain drug curcumin. In vivo pharmacokinetic and biodistribution studies, carried out in rodent model (Sprague–Dawley rats), showed about 1.7-fold absolute bioavailability of curcumin cocrystal micelles and a significantly high brain distribution even beyond 6 h after dosing. Thus, according to the authors’ conclusions, the research demonstrated that curcumin cocrystal micellar nanocarriers enhanced bioavailability, obtained higher brain uptake, and delayed clearance.

Lungare et al. prepared mesoporous silica nanoparticles designed for nose-to-brain drug delivery and containing curcumin and chrysin [26]. Mesoporous silica nanoparticles are a class of biocompatible nanomaterials that is gaining more and more attention for drug delivery because these nanomaterials have low toxicity, high porosity, and high drug loading capacity [54,55]. Chrysin is a polyphenolic flavone present in honey and propolis with antioxidant properties and, similar to curcumin, it is characterized by low water solubility, poor oral bioavailability, and limited BBB penetration. Mesoporous silica nanoparticles with a size of about 220 nm were prepared and then loaded with the two antioxidants. Differential scanning calorimetry (DSC) and thermogravimetric analysis (TGA) were used for their in vitro characterization. The nanoparticles were shown to be non-toxic to olfactory neuroblastoma cells OBGF400 (porcine olfactory bulb neuroblastoma cell line). The active uptake of the nanoparticles into olfactory bulb neuronal cells was demonstrated using mesoporous silica nanoparticles loaded with fluorescein isothiocyanate. These preliminary results led the authors to state that these nanocarriers loaded with curcumin or chrysin can be potentially useful for the nose-to-brain targeting of antioxidants.

Vaz et al. studied nasal lipid nanocarriers containing curcumin to achieve brain targeting [27]. The lipid nanocarriers were obtained with the hot solvent diffusion technique. The nanocarriers were characterized for their morphology and physicochemical properties, and in vitro drug release tests were made. Curcumin permeation/retention was studied in Franz-type diffusion cell through porcine nasal mucosa. Confocal laser scan and histopathological studies were carried out. The nanoparticles had sizes in the range of 18–44 nm and were characterized by negative zeta potential. Curcumin permeation through the porcine nasal mucosa was higher with respect to the permeation of the drug alone. No toxicity on the porcine nasal mucosa was found in the histopathological analysis. The results led the authors to claim that the improvement of the permeation of the drug through the porcine nasal mucosa obtained thanks to the encapsulation of curcumin into the lipidic nanoparticles encourages further studies for the development of nose-to-brain formulations of curcumin based on this kind of nanocarriers.

Zhuang et al. proposed exosomes to encapsulate curcumin [28]. Exosomes are nanovesicles secreted by cells of the immune system such as macrophages, T cells, and dendritic cells. Several researchers showed that they can be utilized as possible drug delivery carriers, and it has been reported that the loading of drugs into exosomes leads to an increase of the circulation time [56]. Exosomes containing curcumin determine an increase of solubility, stability, and bioavailability of the antioxidant. The following inflammation-mediated disease murine models were used: a lipopolysaccharide (LPS) induced brain inflammation model, an experimental autoimmune encephalitis (EAE), and a GL26 brain tumor model. The nose-to-brain administration of the curcumin-loaded exosomes to the mice led to a protection from the LPS-induced brain inflammation and from the progression of induced EAE. It also determined a delay in the growth of the GL26 tumor model. The in vivo distribution studies (on C57BL/6j mice) showed that nasal administered exosomes were taken up mainly by microglial cells. According to these results, the authors claim that exosomes can be considered the base for a potential alternative therapy of brain diseases with inflammatory manifestations. However, they also conclude that further studies are needed to understand the way of exosome transportation from the olfactory region to the brain and the mechanisms of exosome clearance.

The general consideration that can be made about the use of curcumin is that the potential of this substance is high despite the problems connected to this molecule, some of which are strongly challenging from a formulative/biopharmaceutical point of view, such as low solubility and consequent low oral bioavailability. However, the results obtained are encouraging about the potential use in the nose-to-brain route thanks to the design of innovative formulations such as nanocrystals, for example. Unfortunately, to our knowledge no clinical studies concerning the nasal route are currently underway.

### 2.2. Resveratrol

**Figure 3:**
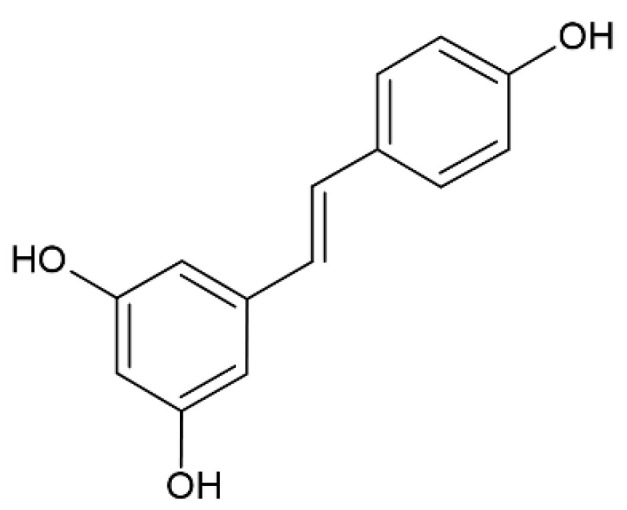
Resveratrol.

Resveratrol (3,5,4′-trihydroxystilbene) (Figure 3) belongs to the group of plant-derived polyphenol compounds, and it is present in different kinds of vegetables and fruits, especially in red grapes. It is characterized by antioxidant/anti-inflammatory properties [57]. Researchers reported its potential in preventing Alzheimer’s disease via the suppression of the formation and aggregation of the Aβ peptide associated with this kind of disease [58].

Resveratrol protects the cells from the oxidative damage by its antioxidant capability based on the scavenging of free radicals [59].

However, its therapeutic efficacy is limited, owing to its low aqueous solubility and extensive intestinal and hepatic metabolism (so-called “first-pass effect”), which determine a poor oral bioavailability [60]. A study concerning a randomized, double-blind, trial of resveratrol carried out on individuals with mild to moderate Alzheimer’s disease showed that resveratrol, orally administered once daily, and its major metabolites penetrate the BBB [61]. However, the intensive metabolization of resveratrol administered orally strengthens the rationale for the intranasal use of this molecule.

Hao et al. prepared a nasal formulation incorporating resveratrol-loaded nanosuspensions into an ion-activated in situ gel [29]. Resveratrol nanosuspensions were prepared using the technique of anti-solvent precipitation and were dispersed into a solution of deacetylated gellan gum. Deacetylated gellan gum is a polysaccharide with linear repeating tetra-saccharide units that forms transparent gels in the presence of cations, such as calcium, present in the nasal mucosa mucus, which cross-link the three-dimensional network of the linear polymer. Particle size, morphology, and stability studies were performed for the in vitro characterization of the nanosuspensions. A rheological evaluation was made on the gel because a favorable rheology ensures that in situ nasal formulations reach olfactory and trigeminal regions and lead to a gel layer that determines an extended retention time and an enhanced drug bioavailability. Pharmacokinetic and brain distribution studies were conducted after nasal administrations of resveratrol-loaded nanosuspensions based on the in situ gel and compared to the intravenous administration of resveratrol nanosuspensions. The animal studies were carried out using Kunming albino mice. The formulation containing 0.6% *w/v* of deacetylated gellan gum showed a favorable gelling ability and the desired viscosity for the intranasal administration. Pharmacokinetics showed a 2.88-times increase of bioavailability in the brain by intranasal in situ gel formulation with respect to intravenous administration. The distribution and localization confirmed the direct nose-to-brain transport, bypassing the BBB. This situ gel formulation containing resveratrol is a promising therapeutic tool that is able to enhance the drug permeability through nasal mucosa and to increase the drug residence time in the nasal cavity.

A nanostructured lipid carrier formulation based on cetyl-palmitate, Capmul MCM, and Acrysol K150, containing resveratrol and designed for nasal administration has been proposed by Rajput et al. [30]. The nanoparticles were prepared by the melt emulsification–probe sonication method. A mean particle size of 132 nm was determined by dynamic light scattering, and a negative zeta potential (−23 mV) was observed. The fresh nasal mucosa of sheep was used for permeation studies. Male Sprague–Dawley rats were used for in vivo pharmacodynamic studies: the transient memory loss in rats was induced by the administration of scopolamine, using the Morris Water Maze test. The results obtained by the Morris Water Maze test demonstrate that the drug-loaded lipidic carrier, after administration through the nose, could effectively treat Alzheimer’s disease on the contrary of the orally administered resveratrol suspension.

Trotta et al. prepared resveratrol-loaded lipidic microparticles, which were uncoated or coated with chitosan and designed for nose-to-brain administration [31]. The drug-loaded microparticles were obtained by melt emulsification, utilizing for their preparation stearic acid and a surfactant (phosphatidylcholine). The coating with chitosan was obtained with the addition of a chitosan solution to the formed microparticles. The chitosan coating changed the charge of the particle surfaces from a negative zeta potential value for the uncoated particles to positive values for coated particles. Permeation studies were made using human NCM460 cell monolayers that were used as an epithelial model, because this kind of cell is able to create monolayers that are tightly connected. The permeability of the drug through the layer of cells is higher in the case of chitosan-loaded particles, according to the well-known properties of chitosan that determine the opening of the tight junctions. In vivo studies were carried out on Male Wistar rats and showed no resveratrol in the rat cerebrospinal fluid (CSF) after an intravenous infusion of the polyphenol while the intranasal administration of the chitosan-coated microparticles produced an increase of bioavailability of resveratrol in the CSF. The authors claim that this increase in the bioavailability in CSF can determine an important neuroprotective effect for the treatment of neurological disorders.

Resveratrol bioavailability has been improved by Salem et al. developing an intranasal transferosomal mucoadhesive gel [32]. Transferosomes are flexible/deformable vesicles constituted by phospholipids: their deformability allows them to squeeze between the cells, improving the permeation of drugs [62]. The reverse evaporation–vortexing sonication technique was used for the preparation of resveratrol-loaded transferosomes that were in vitro characterized for globule size, encapsulation efficiency, and in vitro drug release and then incorporated into mucoadhesive selected gels based on Carbopol 934: ex vivo permeation (nasal septum of Wistar albino rats), histopathological examination for nasal mucosa tolerability, and in vivo pharmacokinetic studies on animals (male Wistar albino rats) were carried out. Histopathological studies showed the safety of the optimized formulation, while the in vivo studies showed bioavailability enhancement by passing intestinal and hepatic metabolism. The same authors prepared a resveratrol formulation constituted by a nasal nano-emulgel based on Carbopol 934 and Poloxamer 407 [63].

The literature also contains a couple of examples in which the nose-to-brain formulation is characterized by a combination of resveratrol with another antioxidant. It is interesting to note that in both cases, the formulation proposed is a nanoemulsion, demonstrating the increasing importance of this kind of formulation in the field of nanomedicine, due to its flexibility.

The first example is the combination of resveratrol and vitamin E, which was used for the preparation of nanoemulsions by Pangeni et al. [33]. The nanoemulsions were obtained by a spontaneous emulsification technique, which was followed by high-pressure homogenization. The formulations were in vitro characterized by globule size, surface morphology, zeta potential, viscosity in vitro release, and ex vivo permeation. The antioxidant activity studied with a DPPH assay showed high scavenging efficiency; the significant antioxidant activity for the nanoemulsions is attributed by the authors to the synergistic antioxidant activity of resveratrol and vitamin E together. Pharmacokinetic studies carried out on Wistar rats showed the high concentration of the drug in the brain following nasal administration of the nanoemulsions. The presence of vitamin E in the formulation is certainly a positive element in the preparation of a formulation with antioxidant activity for the brain, according to many data found in the literature: some studies showed low levels of vitamin E in the blood of Alzheimer’s patients [64], and the level of vitamin E in the plasma has been found to be significantly lower in Parkinson’s disease patients [65].

The second example is the combination of resveratrol with curcumin: Nasr prepared a mucoadhesive nanoemulsion based on hyaluronic acid, co-encapsulating the two polyphenols [34]. The nanoemulsions were prepared by the spontaneous emulsification technique. The characterization in vitro showed a spherical morphology, negative zeta potential, a particle size of about 115 nm, and a significant mucoadhesive strength. Nanoemulsions were able to maintain the antioxidant properties of the two polyphenols, protecting them from degradation. Ex vivo permeation tests through sheep nasal mucosa showed the capacity of the nanoemulsions to determine a comparable permeation flux of both polyphenols through the mucosa, while in vivo tests showed an increase of their amounts in the brain: about 7- and 9-folds increase in AUC0-7 h for resveratrol and curcumin, respectively, with respect to aqueous solutions.

Analogously to curcumin, resveratrol has a big potential, but also in this case, there are remarkable problems from a formulative/biopharmaceutical point of view, which are due not only to low solubility but also to large enzymatic degradation and consequent poor bioavailability. As in the case of curcumin, no clinical studies concerning the nasal route and involving resveratrol formulations are currently underway, to our knowledge.

### 2.3. Naringenin

**Figure 4:**
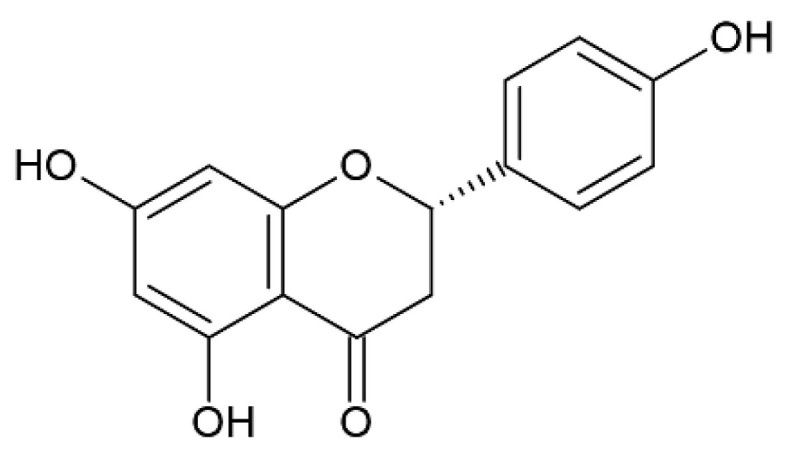
Naringenin.

Naringenin (5,7,4-trihydroxyflavanone) (Figure 4) is one of the most important flavonoids, present in the tomatoes, grapefruits and in some edible fruits, such as *Citrus* species, with anti-inflammatory action and a remarkable antioxidant activity.

Naringenin shows scavenging properties for oxygen-free radicals and also metal chelating action, but it is characterized by low solubility in water and gastrointestinal degradation, and it has therefore very poor bioavailability [66]. In vivo studies carried out in several animal models have shown that naringenin is active against Parkinson’s disease when systemically administered; however, the clinical development of naringenin formulations is limited owing to its low bioavailability [67,68].

Md et al. prepared and characterized naringenin-loaded chitosan nanoparticles for nose-to-brain delivery to obtain a formulation with potential antioxidant and neuroprotective effects for the treatment of Parkinson’s disease [35]. The aim of the presence of chitosan was to make the formulation mucoadhesive. The nanoparticles were obtained using ionic gelation method. In vitro drug release of nanoparticles was studied by placing them in an activated dialysis bag in a phosphate buffer solution. The cellular uptake, cytotoxicity, and neuroprotective action of drug-loaded chitosan nanoparticles were studied using the SH-SY5Y cell line. Ex vivo nasal permeation studies of drug-loaded nanoparticles and of the drug solution were carried out using goat nasal mucosa with Franz diffusion cells. The average particle size was about 88 nm, the zeta potential was about 15 mV, the entrapment efficiency was more than 90%, and the 24 h in vitro release profile was about 55%. The cellular uptake of the nanoparticles was confirmed by the fluorescence microscopy. According to the authors’ opinion, animal studies are necessary to demonstrate that naringenin-loaded chitosan nanoparticles administered by the nose-to-brain route could be effective in the treatment of Parkinson’s disease.

Brain ischemia is the second largest neurological disease, and the oxidative stress is known to be one of the most important factors influencing the development of cerebral ischemia. A poloxamer-chitosan-based naringenin nanosized formulation has been designed and prepared by Ahmad et al. using brain targeting for a possible treatment of cerebral ischemia [36]. Firstly, the naringenin nanoemulsion was prepared and then chitosan and Poloxamer-407 (a thermosensitive hydrogel forming agent) have been added to obtain a thermosensitive in situ/mucoadhesive gel formulation. The aim of the research was to obtain an intranasal formulation able to target the drug to the brain from the nose, with mucoadhesive properties to improve retention time, and to be used in the therapy of cerebral ischemia. The characterization of the formulation was made studying the temperature and the time of gelation, the viscosity, the hydrodynamic diameter, the polydispersity index (PDI), and the zeta potential and performing in vitro gel erosion studies. In vivo studies on animals were carried out in Wistar rats involving histopathological, biodistribution, pharmacokinetics and neurobehavioral/pharmacodynamic studies in cerebral ischemia. The biodistribution studies showed a remarkable enhancement of brain bioavailability of naringenin after the intranasal administration of the nanoemulsion, with respect to intravenous administration of a drug solution and a corresponding decrease of the systemic side effects. For this reason, it can be proposed as a formulation that can be easily used for cerebral ischemia treatment. This work appears to be particularly of interest, since the results, although preliminary, are particularly promising, also given the severity of the neurological pathology, and in this case, clinical studies are highly desirable. These studies also confirm the importance of nanoemulsions as formulations in the field of the brain delivery in nanomedicine, especially as carrier of drugs with problems of poor water solubility.

Nanoemulsions containing a combination of naringenin and vitamin E, for the nose-to-brain administration, have been prepared as a potential treatment of Parkinson’s disease [37]. Previous studies have shown that the administration of vitamin E with an additional antioxidant can be useful in the treatment of neurodegenerative disorders (Alzheimer’s disease) [33]. Ex vivo permeation studies were carried out with a Franz diffusion on freshly isolated goat nasal mucosa, while pharmacokinetic studies were carried out on animals, using Wistar rats. The conclusions of this research were that the nasal administration of narigenin/vitamin E nanoemulsions enhanced the uptake of narigenin into the brain and improved its bioavailability, avoiding systemic circulation and first-pass metabolism. This effect of vitamin E is interesting and worthy of further studies.

### 2.4. Genistenin

**Figure 5:**
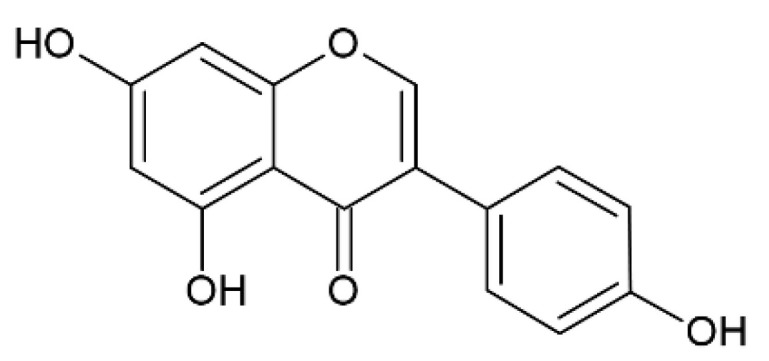
Genistein.

Genistein (Figure 5) is an isoflavonoid phytoestrogen that is well-known for its antioxidant and neuroprotective properties [69].

Despite encouraging preliminary results, low water-solubility and consequent poor oral bioavailability limit its application in clinical studies. Therefore, efficient delivery systems able to cross the BBB must be designed and studied for this kind of drug, and the intranasal route is particularly suitable for this purpose. Rassu et al. prepared and characterized genistein-loaded chitosan nanoparticles for nose-to-brain application [38]. The nanoparticles were achieved by the ionic gelation technique, using sodium hexametaphosphate as a new cross-linker. The systems were characterized in vitro, and ex-vivo permeability was studied using swine nasal mucosa in comparison with a suspension of the drug in a phosphate buffer solution at pH 6.5. The cytotoxicity was tested using PC12 cells. The nanoparticles obtained were small (mean diameter of about 200–300 nm) and homogeneous, and they improved the penetration of genistein through the nasal mucosa with respect to genistein pure drug. The dispersions of the nanoparticles did not show remarkable changes in terms of cell viability and apoptotic events on PC12 cells.

### 2.5. Kaempferol

**Figure 6:**
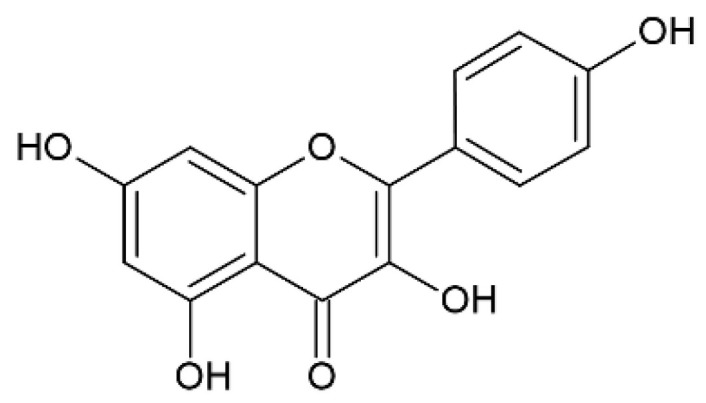
Kaempferol.

Kaempferol (3,4′,5,7-tetrahydroxyflavone) (Figure 6) is a flavonol that can be found in some plants, such as edible plants (broccoli, strawberries, apples, and beans) and medicinal plants (*Rosmarinus officinalis*, *Ginkgo biloba*, *Aloe vera*, *Hypericum perforatum* L., *Crocus sativus* L.).

This drug has antioxidant, anti-inflammatory, and neuroprotective properties, and some studies have shown that it presents also anti-cancer activities in various types of cancer cells, including glioma cells [70,71,72]. However, low solubility in water and consequent poor bioavailability represent a limit for its application in the clinical field [73].

Gliomas are a deadly type of intrinsic brain tumor, and despite the application of multimodal therapy (neurosurgical resection followed by radiotherapy and chemotherapy), the median overall survival of the patients is not favorable [74,75]. Moreover, one of the main factors that impairs the pharmacological treatment is the presence of the BBB. Colombo et al. prepared kaempferol nanoemulsions, with and without chitosan, to study their potential for nose-to-brain application following nasal administration and to study their antitumoral activity against glioma cells (C6 rat glioma cell line) [39]. The nanoemulsions were obtained using the technique of high-pressure homogenization and were characterized for their morphology, drug content, zeta potential, globule size, viscosity, pH, and mucoadhesive strength. An ex vivo diffusion study of formulations was determined using a Franz diffusion cell through a freshly isolated pig nasal mucosa. The in vivo quantification of kaempferol in rat brains was carried out using Wistar rats (*n* = 4). The results of ex vivo diffusion studies showed that the chitosan nanoemulsion is characterized by higher permeation across the mucosa with respect to the formulation without chitosan. Histopathological examinations suggested that nanoemulsions were safe for the nasal mucosa and able to preserve the drug antioxidant capability. Chitosan nanoemulsions significantly enhanced the quantity of drug found in the brain of the rats following the nasal administration (5- and 4.5-fold higher than with free drug and nanoemulsion without chitosan, respectively). Furthermore, chitosan nanoemulsions reduced C6 glioma cell viability through induction of apoptosis to a greater extent than either free kaempferol and the emulsions without chitosan. According to the results obtained, the authors’ conclusion is that the preparation of chitosan nanoemulsions can be considered a promising technique to obtain the nose-to-brain targeting of kaempferol and hence provides a potential candidate for preclinical studies on gliomas.

### 2.6. Rutin

**Figure 7:**
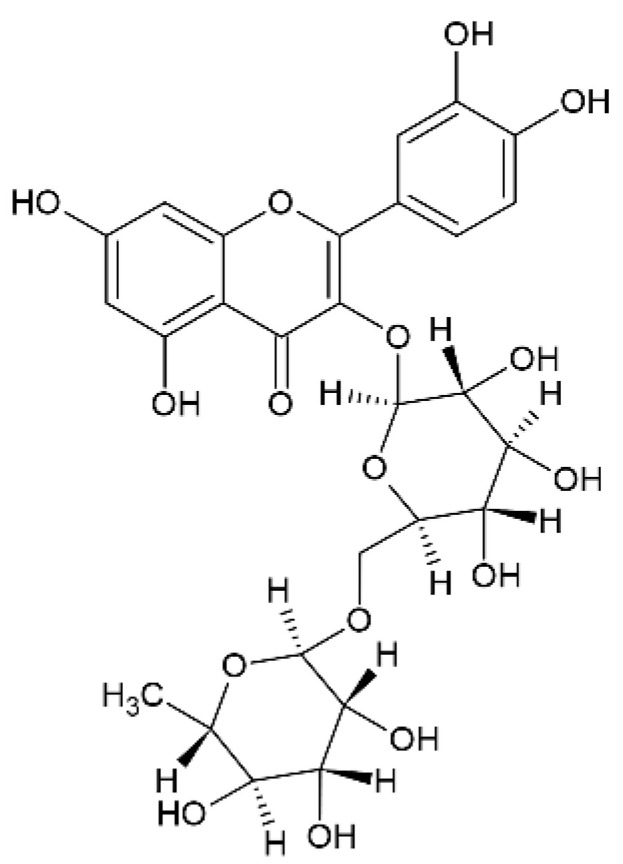
Rutin.

Recent studies have shown that rutin (Figure 7), a lipophilic natural antioxidant drug present in *Carpobrotus edulis* and *Ruta grave-olens*, can be a therapeutic tool for the treatment of cerebral ischemia, but it has problems due to its low water solubility and hence low bioavailability, enzymatic degradation in the gastrointestinal tract, and extensive hepatic first-pass metabolism [76].

Ahmad et al. obtained rutin-loaded chitosan nanoparticles via the ionic gelation technique, which were designed for the nose-to-brain administration [40]. Chitosan of medium molecular weight was used. The nanoparticles were characterized in vitro in terms of particle size and zeta potential, morphology (scanning electron microscopy), loading capacity, encapsulation efficiency, and in vitro release. Ex vivo permeation studies were carried out on fresh nasal mucosa excised from the nasal cavities of goats. A porcine mucin suspension was used for the determination of mucoadhesive strength of chitosan nanoparticles. In vivo studies were performed on Wistar rats (*n* = 6). Nanoparticles having a size lower of <100 nm and with an encapsulation efficiency of about 85% were obtained. The release profile of rutin from chitosan nanoparticles showed a slow/sustained release pattern achieved with the possible contribution of both diffusion-controlled and swelling-controlled mechanisms. Rutin-loaded chitosan nanoparticles showed more permeation compared to the pure drug solution: according to authors’ opinion, the enhanced permeation for rutin loaded in the chitosan nanoparticles may be due to the interaction of positively charged amino groups of the polymer with negatively charged sites present on the cell membranes. Furthermore, chitosan nanoparticles exhibited excellent mucoadhesive strength. Pharmacokinetics, biodistribution, and brain targeting efficiency studies showed enhanced bioavailability for rutin in the brain as compared to the intravenous administration. According to these results, the authors claimed that chitosan played an important role for its mucoadhesive and penetration enhancer properties. Rutin-loaded chitosan nanoparticles, nasally administered, are a possible non-invasive brain drug delivery system for the possible treatment of cerebral ischemia.

### 2.7. Thymoquinone

**Figure 8:**
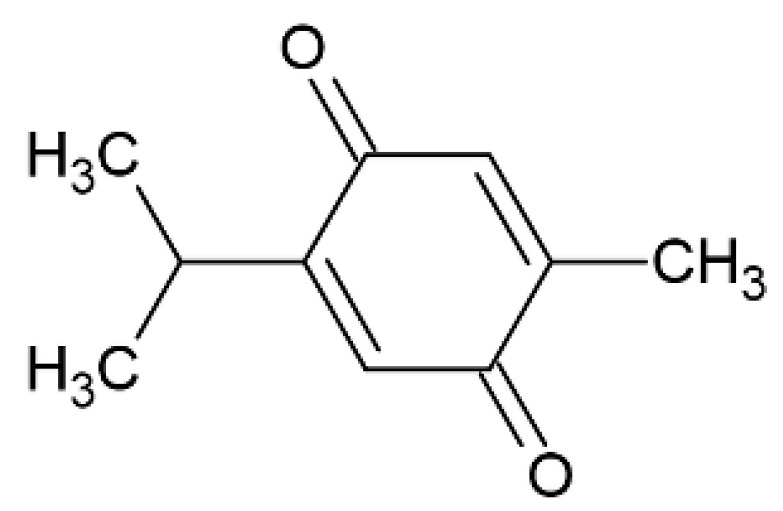
Thymoquinone.

As previously reported, antioxidants can be utilized as potential tools to prevent and treat cerebral ischemia. In vitro and in vivo studies have shown that thymoquinone (Figure 8), a liposoluble benzoquinone of natural origin and component of the volatile oil from the seeds of *Nigella sativa*, can be used in the therapy of cerebral ischemia [77]. This compound is characterized by antioxidant, anti-inflammation, and anticancer properties; however, it has poor solubility, extensive metabolism, and rapid elimination with consequent poor bioavailability.

Mucoadhesive nanoemulsions based on chitosan and designed for nasal administration were obtained by the ionic gelation technique, using oleic acid as oil [41]. The formulation was characterized in vitro for particles size, encapsulation efficiency, and zeta potential. Ex vivo permeation studies were made across goat nasal mucosa. In vivo pharmacokinetic and biodistribution studies were carried out on Wistar rats. Enhanced drug permeability from the mucosa and improved bioavailability into the brain showed that these nanoemulsions can be potentially useful in a therapy of cerebral ischemia using thymoquinone. This work represents a further confirmation of the great potential of nanoemulsions: their hydro/lipophilic nature is optimal for carrying poorly soluble drugs; furthermore, they can also be made mucoadhesive, which is a very important property for a nasal formulation.

### 2.8. Pyrrolidine Dithiocarbamate

**Figure 9:**
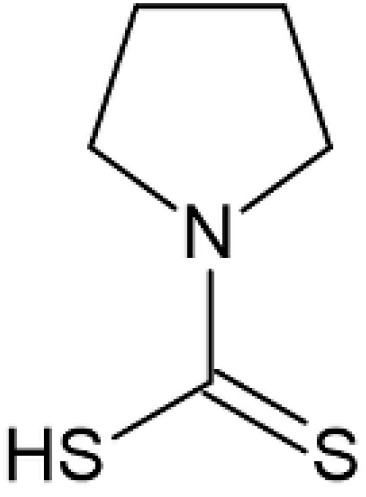
Pyrrolidine dithiocarbamate.

Pyrrolidine dithiocarbamate (Figure 9) is an antioxidant and anti-inflammatory agent [78].

It has been reported that it can reduce focal brain ischemic injury in young and adult rats [79] and in neonatal rats [80] after intraperitoneal administration. It is an interesting low molecular weight product showing also relatively low costs [42]. Wang et al. considered that in the management of birth asphyxia, the nasal administration of this drug can be a valid alternative to the intraperitoneal administration [42]. The intranasal application of the drug was carried out in the form of nasal drops in saline solution. A rat model of neonatal brain ischemic injury was used, involving 7-day-old male and female Sprague–Dawley rats, because both genders can suffer from birth asphyxia. The results showed that the intranasal administration of pyrrolidine dithiocarbamate provides a neuroprotective effect, probably due the direct reduction of inflammation and oxidative stress. Therefore, the nose-to-brain targeting of this drug can have the potential to achieve neuroprotection to human neonates after birth asphyxia. Chabicovsky et al. demonstrated that significant amounts of pyrrolidine dithiocarbamate are present in the brain after nasal application [78].

### 2.9. Rosmarinic Acid

**Figure 10:**
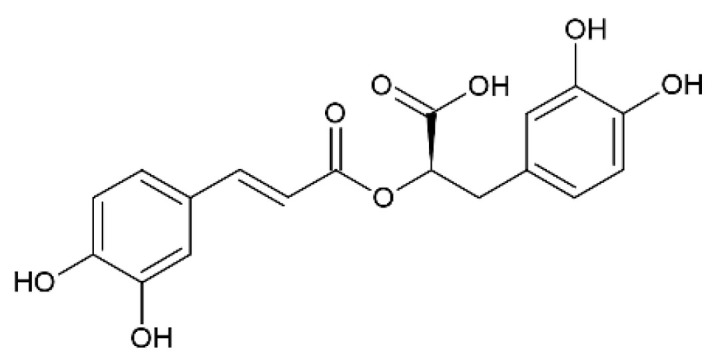
Rosmarinic acid.

Rosmarinic acid (Figure 10) is a polyphenol (ester of caffeic acid and 3,4-dihydroxyphenyllactic acid) isolated from rosemary (*Rosmarinus officinalis* L.), characterized by strong anti-inflammatory properties [81].

Recently, Rahbardar et al. demonstrated the ability of rosmarinic acid to modulate neuro-inflammation using a neuropathic pain murine model following the administration in the neuro-inflammatory environment [82]. In particular, these authors showed that rosmarinic acid has a specific anti-inflammatory effect on neuro-inflammations through the reduction, in the treated animals, of many inflammatory and oxidative markers such as cyclooxygenase 2 (COX2), prostaglandin E-2, and matrix metalloproteinase 2, and for these reasons, they suggested that this compound could be a good candidate for the treatment of inflammatory neurological disorders. The current literature shows that many of the in vivo studies that regard the use of rosmarinic acid for neuroprotection have been carried out using intragastric administration [81]. However, there are in the literature several pharmacokinetic studies demonstrating its poor oral bioavailability due to the poor water solubility of this compound [81,83]. Furthermore, another important reason for poor bioavailability is the low stability of rosmarinic acid following oral administration [84]. For all these reasons, rosmarinic acid can be considered a good candidate for the nose-to-brain route.

Bhatt et al. prepared solid lipid nanoparticles (SLNs) exhibiting a particle size of about 150 nm, containing rosmarinic acid and designed for the treatment of Huntington’s disease [43]. SLNs based on glyceryl monostearate as a lipid were prepared using a hot homogenization technique. A murine Huntington’s disease model was used and demonstrated the ability of SLNs to attenuate the motor and locomotor deficits and the striatal oxidative stress. The pharmacokinetic studies on Wistar rats compared the nasal and intravenous routes and showed a higher brain concentration when rosmarinic acid was administrated through the nasal route. According to the good results obtained, the authors claim that rosmarinic acid-loaded SLNs administered through the non-invasive nose-to-brain drug route can be considered a promising potential strategy for the treatment of Huntington disease.

Chitosan-coated nanoemulsions containing rosmarinic acid and designed for nasal delivery have been recently proposed [44]. The nanoemulsions were prepared with medium chain triglycerides and egg lecithin (lipid phase) using an oil-in-water spontaneous emulsification technique and had a mean size of about 258 nm. Then, the emulsion was coated with chitosan. The nanoemulsions showed long-lasting permeation through porcine nasal mucosa, with a good safety behavior demonstrated using the MRC-5 cell line (normal human lung fibroblasts). The results obtained showed that this kind of mucoadhesive formulation may be suitable for neuroprotective therapies based on the administration of rosmarinic acid through the nose-to-brain route.

### 2.10. Melatonin

**Figure 11:**
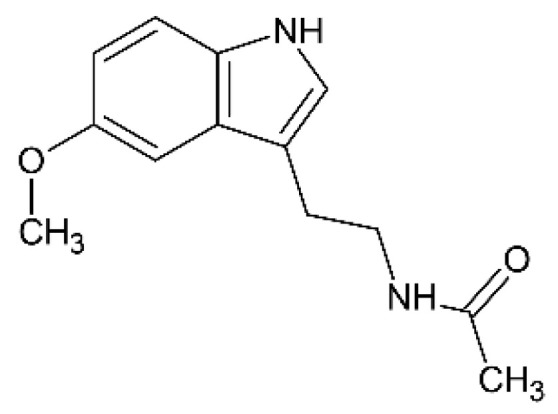
Melatonin.

Melatonin (Figure 11) is a neurohormone secreted by the pineal gland that plays an important role in the synchronization of the rhythms of the body with night and day cycles and for these reasons its use is well-established in the management of sleep disorders, jetlag, and migraine headaches [85]. Several works reported that melatonin has neuroprotective and antioxidant properties against oxidative injury mediated by amyloid β-protein in vitro [86]. Other studies demonstrated that melatonin inhibits the formation of beta-sheets and Aβ fibrils [87], and it has neuroprotective effects in vivo in transgenic mice [88]. Furthermore, it is reported that melatonin improves the activity of enzymes that metabolize active oxygen species to inactive products [89].

However, melatonin is characterized by a very low oral bioavailability related to an extensive first-pass hepatic and its remarkable gut-wall metabolism [90]. According to the Biopharmaceutics Classification System, melatonin is a class II drug, with a very short half-life and minimal and variable bioavailability [91].

Babu et al. prepared gel suspensions based on Carbopol 934P or carboxymethyl cellulose (medium viscosity grade), containing melatonin, and intended for nose-to-brain delivery [45]. The drug was subjected to micronization by a ball-milling process and then added to a gel prepared with 0.125% Carbopol or 1% CMC and 0.1% *w/v* Tween-80. The rheological behavior of the gels was studied using a Brookfield viscosity meter. The studies of permeability were carried out using three-dimension bronchial/tracheal (human-derived) epithelial cell cultures (EpiAirway^™^); these same cell cultures were already utilized as permeation barriers of nasal membranes in previous investigations [92,93,94]. Male Wistar rats were used for the in vivo tests in which the nasal administration of the gel was compared to intravenous route. Carbopol and CMC gels determined a high permeability of melatonin across EpiAirway^™^. The brain and olfactory bulb levels of melatonin after the nasal administration were about 9 and 7 folds higher for Carbopol and CMC, respectively, than that of intravenous melatonin in the rats. These studies showed that melatonin is transported into the brain via the olfactory bulb after the nasal administration and, according to the authors’ opinion, indicated that melatonin can have potential neuroprotective roles in the therapies of Alzheimer’s and Parkinson’s diseases.

Furthermore, melatonin shows antitumor activity in a wide range of tumors in vitro and in vivo [95]. A previous work described an increase of glioma patient survival using radiotherapy plus melatonin compared with radiotherapy alone [96]. Martin et al. showed that melatonin inhibits C6 glioma cell proliferation [97]. Pan et al. recently reported the possible use of melatonin for the treatment of the chemoresistance of glioblastoma [98]. All these studies make the rational base for the work of Ribeiro de Oliveira et al. that prepared polycaprolactone nanoparticles containing melatonin and designed for the nose-to-brain treatment of glioblastoma [46]. The drug-loaded nanoparticles were prepared by a nanoprecipitation technique, using polycaprolactone (average mw 80,000) as a polymer. In vitro release studies were carried out with dialysis tubes. U87MG cells (human glioblastoma cell line) and MRC-5 cells (human pulmonary fibroblasts) were used for the tests of cellular uptake of nanoparticles evaluation following the incubation of the nanoparticles. Cytotoxicity against U87MG glioblastoma cells and MRC-5 non-tumor cells was determined. The nanoparticles showed a strong efficacy against U87MG cells, resulting in IC50 about 2500 folds lower than that of the free drug. No cytotoxic effect was observed against non-tumor cells. In vivo studies were carried out on rats: the nasal administration of the drug-loaded nanoparticles determined a higher AUC in the brain compared to the administration of free drug by either intranasal or oral route.

Due to its well-known neuroprotective and antioxidant properties, melatonin is a molecule of great interest. The neuroprotective properties in the case of radiotherapy and its possible use in the case of chemoresistance in glioblastoma are certainly worthy of further studies and insights. The nose-to-brain administration of appropriately formulated melatonin could be a good opportunity for the prevention of relapses in the maintenance therapy.

### 2.11. Catalase

Catalase can be considered one of the most potent antioxidants in nature: it is a redox enzyme that deactivates one million free radicals per second per molecule of catalase in a reaction cycle [99]. As previously reported, Parkinson’s disease is associated with brain inflammation and, in the case of this disease, samples from brains showed reduced levels of catalase [7]. A deficiency or malfunction of catalase has been recently related to the pathogenesis of age-associated degenerative diseases such as Parkinson’s disease and Alzheimer’s disease [100]. However, BBB limits the passage of this protein into the brain, and this impaired the development of any therapeutic approach involving this enzyme.

Haney et al. showed that macrophages preloaded with nanoformulated catalase release exosomes with incorporated catalase [99]. Starting from this observation, these authors developed a formulation based on exosomes containing catalase and proposed it for Parkinson’s disease therapy [47]. Catalase from bovine liver was used, and the loading of the enzyme was carried out into naïve exosomes ex vivo comparing different methods for incorporation: incubation at room temperature with or without saponin permeabilization, freeze–thaw cycles, sonication, or extrusion of exosomes in the presence of catalase. The formulations were characterized regarding size, catalase-loading efficiency, release, and antioxidant activity. The size of catalase-loaded exosomes was determined by Dynamic Light Scattering (DLS) and Nanoparticle Tracking Analysis (NTA): particles of naked catalase showed a size of about 9.5 nm, which is close to the theoretical size of a single protein (10.5 nm) as calculated from the molecular mass of the enzyme. The average size of empty exosomes was about 100 nm, while the size of catalase-loaded exosomes was about 100–200 nm. To study the capacity of exosomes to target catalase to inflamed brain tissues, confocal imaging studies were carried out using a Parkinson’s disease mouse model. To induce brain inflammation, C57BL/6 mice were intracranially injected with 6-hydroxydopamine (6-OHDA). Twenty-one days later, at the inflammation peak, exosomes labelled with a lipophilic fluorescent dye, 1,1′-dioctadecyl-3,3,3′,3′-tetramethylindo-carbocyanine perchlorate (DIL), were administered to the mice through intranasal or intravenous routes. Mice were euthanized four hours later, perfused, and brain slides were studied by confocal microscopy. The results showed that the amounts of exosomes delivered upon the intranasal administration was greater than those administered through intravenous injection. The images showed the distribution of exosomes throughout the brain and especially in the cerebral frontal cortex and cerebellum. To determine in which cells of the brain exosomes accumulate, the authors prepared brain slides co-stained with different cell markers and found that exosomes are mostly localized into neighboring neurons, microglia and partially close to endothelial cells. Therefore, the authors claim that the exosome-mediated delivery of catalase to neurons and microglia in the inflamed brain may likely result in ROS degradation and neuroprotection.

What is most striking from the examination of this literature is the fact that even molecules of considerable size such as enzymes can be conveniently formulated and administered through the nose-to-brain pathway.

## 3. Final Remarks

In the literature, many studies show that ROS generation and oxidative stress play an important role in the pathogenesis of neurodegenerative disorders (Alzheimer’s, Parkinson’s diseases) and of the damages present in the transient cerebral ischemia.

The development of neuroprotective formulations containing antioxidants as a potential innovative tool for the therapeutic treatment in neurological diseases represents an important goal for the current neuropathological research. However, there are different bioavailability problems still to be overcome in case of systemic administration: the passage of the BBB that represents a remarkable obstacle to the brain targeting, the low solubility in water of many antioxidants, their instability in the gastrointestinal environment, and possible metabolization.

For these reasons, formulative and preclinical studies are carried out in animal models using innovative formulations such as polymeric nanoparticles, nanocrystals, nanoemulsions, and exosomes, which are administered through the nose-to-brain route.

This overview shows that excipients play an important role in the design of the nose-to-brain formulations. As shown by the literature, chitosan certainly plays a major role both for the penetration enhancer and for the mucoadhesion properties. Often, the formulations proposed involve the addition of a mucoadhesive polymer to overcome the problem of nasal clearance [31,35,36,38,39,40,41,44,101,102]. Lipidic excipients are frequently used both for the preparation of lipidic nanoparticles [27,30,31,43] and as components of nanoemulsions [33,34,36,39,41]. The importance of the lipidic excipients in the design of intranasal formulations is confirmed by the literature [103], and it is probably based on the improvement of solubilization and loading of hydrophobic molecules and on the hydrophilic/lipophilic nature of the barriers present in the nose-to-brain pathways.

Moreover, nose-to-brain nanoemulsions allow the administration of combinations of different actives. Exosomes represent a new strategy to bypass of BBB even for large molecules such as enzymes.

The promising results reported in these studies do not allow equalizing these findings to human use. They must be confirmed by further experiments, and therefore, additional research work is still needed to translate all these experimental results from animal models to the clinical application for humans. It is worthwhile mentioning a clinical study involving the intranasal administration of glutathione in Parkinson’s disease based on the evidence that excessive free radical formation and depletion of glutathione in the brain are connected to the development of this pathology [104]. The aim of this study is the evaluation of the safety, tolerability, and preliminary absorption data of glutathione in volunteers with Parkinson’s disease in a Phase I single ascending dose escalation study.

Hopefully, in the near future, clinical studies will arise involving the antioxidants reported in this review, thanks to the formulative solutions here described to make their administration possible. The use of the nose-to-brain route to overcome pharmacokinetic problems will allow future preclinical and clinical studies to better clarify the effect that antioxidants can have in the CNS.

## Figures and Tables

**Table 1:** Curcumin-loaded formulations used through the nose-to-brain route as a potential treatment of neurological diseases.

Antioxidant	Neurological Disease	Formulation	Reference	Biological Evaluation
Curcumin	Alzheimer’s disease	Cocrystal micelles	[25]	Sprague–Dawley rats
Curcumin/chrysin	Central nervous system (CNS) diseases	Mesoporous silica nanoparticles	[26]	Neuro blastoma cells OBGF400
Curcumin	Alzheimer’s disease	Lipid nanocarrier	[27]	Franz-type diffusion cell with porcine nasal mucosa
Curcumin	Brain inflammatory diseases	Exosomes	[28]	C57BL/6j mice

**Table 2:** Resveratrol-loaded formulations used through the nose-to-brain route as a potential treatment of neurological diseases.

Antioxidant	Neurological Disease	Formulation	Reference	Biological Evaluation
Resveratrol	Alzheimer’s disease	In situ gel (Gellan gum) nanosuspension	[29]	Kunming albino mice
Resveratrol	Alzheimer’s disease	Nanostructured lipid carrier	[30]	Male Sprague–Dawley rats
Resveratrol	Alzheimer’s disease	Chitosan coated lipid microparticles	[31]	Male Wistar rats
Resveratrol	Alzheimer’s disease	Trasferosomes into gel	[32]	Male Wistar albino rats
Resveratrol and vitamin E	Parkinson’s disease	Nanoemulsion	[33]	Wistar rats
Resveratrol and curcumin	Neurodegenerative diseases	Nanoemulsions	[34]	Wistar rats

**Table 3:** Antioxidant-loaded formulations used through the nose-to-brain route as a potential treatment of neurological diseases.

Antioxidant	Neurological Diseases	Formulation	Reference	Biological Evaluation
Naringenin	Parkinson’s disease	Chitosan nanoparticles	[35]	SH-SY5Y cells
Narigenin	Cerebral ischemia	Nanoemulsion	[36]	Wistar rats
Narigenin and vitamin E	Parkinson’s disease	Nanoemulsion	[37]	Wistar rats
Genistein	Alzheimer’s/Parkinson’s diseases	Chitosan nanoparticles	[38]	PC12 cell line
Kaempferol	Gliomas	Nanoemulsion	[39]	C6 rat glioma cell line/Wistar rats
Rutin	Ischemic disease	Chitosan nanoparticles	[40]	Wistar rats
Thymoquinone	Ischemic disease	Nanoemulsion	[41]	Wistar rats
Pyrrolidine dithiocarbamate	Brain hypoxia/ischemia	Drops and spray	[42]	FemaleSprague- Dawley rats
Rosmarinic acid	Huntington’s disease	SLNs	[43]	Wistar rats
Rosmarinic acid	Neurological diseases	Chitosan coated nanoemulsions	[44]	MRC-5 cell line
Melatonin	Alzheimer’s/Parkinson’s diseases	Gel suspension	[45]	Male Wistar rats
Melatonin	Glioblastoma	Polycaprolactone nanoparticles	[46]	U87MG and MRC-5 cells
Catalase	Parkinson’s disease	Exosomes	[47]	C57BL/6 mice
